# Quantitative Shotgun Proteomics Unveils Candidate Novel Esophageal Adenocarcinoma (EAC)-specific Proteins * [Fn FN2]

**DOI:** 10.1074/mcp.M116.065078

**Published:** 2017-06

**Authors:** J. Robert O'Neill, Hui-Song Pak, Erola Pairo-Castineira, Vicki Save, Simon Paterson-Brown, Rudolf Nenutil, Bořivoj Vojtěšek, Ian Overton, Alex Scherl, Ted R. Hupp

**Affiliations:** From the ‡Edinburgh Cancer Research Centre at the Institute of Genetics and Molecular Medicine, Edinburgh University;; §Department of Surgery, Royal Infirmary of Edinburgh;; ¶Department of Human Protein Sciences, Faculty of Medicine, University of Geneva;; ‖Centre for Medical Informatics, Usher Institute of Population Health Sciences and Informatics, University of Edinburgh;; **MRC Human Genetics Unit, Institute of Genetics and Molecular Medicine, Edinburgh University;; ‡‡Department of Pathology, Royal Infirmary of Edinburgh;; §§Regional Centre for Applied Molecular Oncology, Masaryk Memorial Cancer Institute, Brno

## Abstract

Esophageal cancer is the eighth most common cancer worldwide and the majority of patients have systemic disease at presentation. Esophageal adenocarcinoma (OAC), the predominant subtype in western countries, is largely resistant to current chemotherapy regimens. Selective markers are needed to enhance clinical staging and to allow targeted therapies yet there are minimal proteomic data on this cancer type. After histological review, lysates from OAC and matched normal esophageal and gastric samples from seven patients were subjected to LC MS/MS after tandem mass tag labeling and OFFGEL fractionation. Patient matched samples of OAC, normal esophagus, normal stomach, lymph node metastases and uninvolved lymph nodes were used from an additional 115 patients for verification of expression by immunohistochemistry (IHC).

Over six thousand proteins were identified and quantified across samples. Quantitative reproducibility was excellent between technical replicates and a moderate correlation was seen across samples with the same histology. The quantitative accuracy was verified across the dynamic range for seven proteins by immunohistochemistry (IHC) on the originating tissues. Multiple novel tumor-specific candidates are proposed and EPCAM was verified by IHC.

This shotgun proteomic study of OAC used a comparative quantitative approach to reveal proteins highly expressed in specific tissue types. Novel tumor-specific proteins are proposed and EPCAM was demonstrated to be specifically overexpressed in primary tumors and lymph node metastases compared with surrounding normal tissues. This candidate and others proposed in this study could be developed as tumor-specific targets for novel clinical staging and therapeutic approaches.

Esophageal cancer is the sixth leading cause of cancer death worldwide (1) and esophageal adenocarcinoma (EAC) ^1^ has become the predominant histological subtype in western countries (2, 3). In the UK, 95% of patients diagnosed with EAC will die from metastatic disease and the majority are resistant, at presentation, to current platinum-based chemotherapy regimens (4–6).

EAC is frequently associated with both lymphatic and distant metastases yet current staging modalities including computed tomography (CT), positron emission tomography (PET) and endoscopic ultrasound (EUS) are limited in both sensitivity and specificity (5). Surgical resection only benefits patients with localized disease and carries a 40% risk of major morbidity and 2–3% risk of perioperative mortality (7, 8). The development of accurate noninvasive imaging markers of EAC would enhance clinical staging by allowing the specific detection of locoregional and distant metastases, enabling treatment stratification (9).

The normal squamous epithelium-lined esophagus is vulnerable to toxic insult from the esophageal lumen. Indeed, chronic reflux of gastric acid and bile is thought to underlie the development of columnar metaplasia, “Barrett's esophagus”, the precursor lesion of EAC (10). Although the exact molecular mechanisms of Barrett's development and esophageal carcinogenesis remain obscure, the detection and treatment of EAC at an early stage offers the prospect of long term cure with over 80% of patients undergoing surgery for stage I esophageal cancer surviving 5 years (11).

Intriguingly many of the genetic mutations present in EAC have also been demonstrated in nondysplastic Barrett's epithelium raising the possibility that a transcriptional change such as splicing or RNA-editing, or a post-translational modification is responsible for transformation (12). If such a biomarker could be identified this would offer the possibility of earlier diagnosis and more effective treatment.

Characterizing the proteomic changes associated with EAC may also allow novel therapies to be designed. Tumor-specific proteins have been exploited as immunotherapeutic targets in other cancer types by engendering a host response to the cancer (13), in some cases leading to durable responses (14).

To date, no specific markers of EAC have been identified. To identify candidate proteins *de novo*, expression must be measured using untargeted proteomic methods.

Quantitative proteomic methods have now been applied across many cancer tissues. Most previous proteomic studies in EAC, however, have only identified a small number of dysregulated proteins limiting the comparisons that can be made between studies or with other cancers (summarized in Table I). Only one of these previous studies employed a quantitative shotgun proteomic strategy. The authors compared pooled biopsies of EAC, normal esophagus, gastric adenocarcinoma and normal gastric tissue and identified 972 proteins. Although no EAC-specific protein was identified, neutrophil defensin 1, an antimicrobial peptide found in neutrophil granules, was overexpressed in both cancer types relative to normal tissue (15). This may reflect the inflammatory environment associated with these cancers.

**Table I:** Published proteomic studies of OAC

Study author	Patients	Method	Tissue preparation	Squamous samples	Gastric samples	Barrett's samples	ACC samples	SCC samples	Total identifications	Number of proteins dysregulated
Zhao^22^	6	LC-ESI TOF MS, Targeted LC-MS/MS	Fresh frozen biopsies	-	-	6	6	-	-	38 proteins
Yoo^68^	1	LC-ESI TOF MS, Targeted MALDI-MS	Fresh frozen biopsies	-	-	-	1	-	22 proteins	-
Peng^21^	8	2D Gels, Targeted MALDI-MS	Fresh frozen biopsies	2	2	-	8	-	-	23 dysregulated gel spots – 22 proteins identified
Langer^69^	20	2D Gels, Targeted MALDI- and LC-MS/MS	Fresh frozen biopsies				20		-	Data for 4 proteins presented
Quaas^70^	477	MALDI-MSI	FFPE slides from a tissue microarray	-	-	-	300	177	72 spectral features 13 peptides	-
Aichler^71^	23	MALDI-MSI. Targeted LC-MS/MS	Fresh frozen biopsies	-	-	-	23		-	22 spectral features, 6 proteins
Elsner^72^	38	MALDI-MSI. Targeted LC-MS/MS	Fresh frozen biopsies	-	-	11	33			61 spectral features, 6 proteins
Streitz^73^	4	MALDI-MS	LCM of fresh frozen biopsies	-	-	4	4		8 spectral features	
Singhal^15^	53 (iTRAQ)	MALDI-MS iTRAQ LC-MS/MS	Fresh frozen biopsies	30	23		30	-	972 proteins	Not described.

Abbreviations: EAC, Esophageal Adenocarcinoma; ACC, Adenocarcinoma; SCC, Squamous Cell Carcinoma; LCM, Laser Capture Microdissection.

The comparisons between EAC and normal squamous epithelium in published work reveals many dysregulated proteins, some of which represent proteins associated with glandular differentiation and some associated with carcinogenesis. Glandular-associated proteins may be expressed in gastric and intestinal epithelium and may not represent tractable targets for therapy as toxicity because of intestinal epithelial damage would be expected. It is possible that including columnar epithelium-lined gastric tissue along with squamous and EAC tissue may enable the discrimination of proteins that reflect glandular differentiation from those driving carcinogenesis.

Multitissue proteomic profiling has been applied across mouse tissues with relative quantitation using a super-SILAC approach (16). In this study, snap-frozen biopsies from 28 tissue types were subjected to shotgun proteomics with a spike-in, heavy-labeled mixture of all tissues obtained from the SILAC mouse. By comparing the relative expression of proteins across tissues, tissue-specific expression could be highlighted. The esophagus was not included in this profiling effort although gastrointestinal tissues with columnar epithelia showed similar expression patterns (16). This comparative approach has also been employed in a large proteomic study of 30 human tissues by label-free quantification and again tissue-specific expression patterns identified (17).

This biomarker discovery study therefore used a quantitative shotgun proteomic strategy to evaluate protein expression in EAC and adjacent matched normal squamous and gastric tissues from seven patients. By quantifying the relative expression between EAC and normal esophagus and EAC and normal stomach, proteins aberrantly expressed in EAC were identified. The accuracy of this approach was confirmed by immunohistochemistry for multiple candidates and a potential tumor biomarker verified in a cohort of 115 patients with resected EAC and matched normal and metastatic tissues.

## EXPERIMENTAL PROCEDURES

### 

#### 

##### Experimental Design and Statistical Rationale

Fresh frozen biopsies representing macroscopically normal esophagus, normal stomach and esophageal adenocarcinoma tissue were prospectively collected from resection specimens from seven patients undergoing neoadjuvant chemotherapy and attempted curative surgery for locally advanced esophageal and esophagogastric junctional cancer at the Royal Infirmary of Edinburgh between 2010 and 2012. Local institutional ethical and research and development approvals were in place (REC references 06/S1101/16 and 10/S1402/33) (R&D ID 2006/W/PA/01). All patients gave informed consent and participants and their donated samples were de-identified at the time of recruitment. Patients were selected for relative clinical homogeneity with respect to known prognostic variables including lymphatic metastasis and tumor differentiation (18). The clinical characteristics of the cohort are presented in Table II.

**Table II:** Clinical characteristics of patients donating tissue for proteomic analysis

	Patient (Pt)						
	Pt44	Pt46	Pt48	Pt51	Pt53	Pt60	Pt61
Gender	Male	Male	Male	Male	Female	Male	Male
Age	59	67	65	41	52	60	58
Histology	ACC	ACC	ACC	ACC	ACC	ACC	ACC
Location	EGJ Type II	Eso Lower	EGJ Type II	EGJ Type I	Eso Lower	Eso Lower	Eso Lower
Neoadjuvant therapy	2xCF	2xCF	2xCF	2xCF	2xCF	2xCF	2xCF
Surgery	ILE	ILE	ILE	ILE	ILE	ILE	ILE
Tumor Diameter	38 mm	70 mm	40 mm	50 mm	83 mm	52 mm	35 mm
PRM and DRM	>1 mm	>1 mm	>1 mm	>1 mm	>1 mm	>1 mm	>1 mm
Distance to CRM	4.2 mm	0.0 mm	0.0 mm	0.3 mm	3.0 mm	1.0 mm	1.0 mm
Resection	R0	R1	R1	R1	R0	R0	R0
Differentiation	Moderate	Poor	Poor	Poor	Poor	Poor	Poor
LVI	Y	Y	Y	Y	N	Y	Y
Venous Invasion	N	N	N	Y	N	N	N
PNI	N	Y	Y	Y	N	Y	Y
T stage	ypT2	ypT4a	ypT3	ypT3	ypT2	ypT3	ypT3
N Stage	ypN1	ypN3	ypN3	ypN3	ypN1	ypN3	ypN2
Positive nodes	2	8	16	7	1	7	3
Nodes resected	27	18	28	28	23	21	37
AJCC Stage	IIB	IIIC	IIIC	IIIC	IIB	IIIC	IIIB
Mandard TRG	V	V	IV	V	IV	V	V
Alive at analysis	No	No	No	No	Yes	No	Yes
Overall survival	48.1 months	15.3 months	10.9 months	24.1 months	47.6 months (censored)	17.8 months	49.0 months (censored)
Recurrence-free survival	34.8 months	12.6 months	10.1 months	10.9 months	47.6 months (censored)	8.1 months	49.0 months (censored)

Abbreviations: ACC, Adenocarcinoma, 2xCF-2 cycles of Cisplatin and 5-Fluorouracil; ILE, Ivor-Lewis Esophagectomy; mm–millimetre, AJCC, American Joint Committee on Cancer; EGJ, Esophagogastric Junctional Tumour; PRM, Proximal resection margin; DRM, Distal resection margin; CRM, Circumferential resection margin; LVI, Lymphovascular invasion; PNI, perineural invasion, Y-Yes, N-No; CT, Computed Tomography, Mandard; TRG, Tumour Regression Grade; Eso, Esophagus.

At the commencement of this study, no shotgun proteomic data were available for esophageal adenocarcinoma tissue to inform a power calculation for sample size determination. The sample number was therefore based on previous esophageal discovery-phase proteomic studies or studies in similar tissue types (19–22), and the availability of high quality clinical material.

Because of the risk of false-positives because of the small sample size, proposed tumor-specific proteins identified by mass spectrometry were additionally verified by immunohistochemistry (IHC) using cores from archival tumors and matched normal and metastatic tissues from an independent cohort of 115 patients with esophageal or EGJ adenocarcinoma (clinical characteristics in supplemental Table S1).

##### Sample Processing

**Fig. 1.**
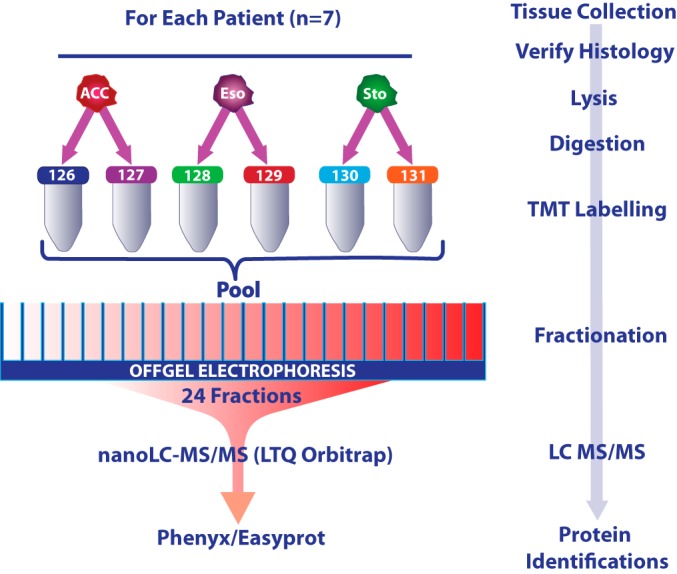
**Summary experimental workflow.** Tryptic peptides from histologically-verified, fresh frozen tissue biopsies were labeled with TMT reporters prior to OFFGEL electrophoresis and tandem mass spectrometry. Abbreviations: ACC, Adenocarcinoma biopsies; Eso-normal squamous esophageal biopsies; Sto-normal gastric biopsies. TMT, Tandem Mass Tags; LC; Liquid Chromatography; MS/MS, Tandem Mass Spectrometry.

The sample processing workflow is summarized in Fig. 1. Fresh tissue biopsies were snap frozen within 30 min of tumor extirpation and maintained in liquid nitrogen or on dry ice until lysis. Frozen sections from each biopsy were reviewed by a consultant histopathologist to confirm the histological diagnosis and, for tumor biopsies, a minimum of 50% tumor cellularity.

The published Filter-Aided Sample Preparation (FASP) method was adapted for protein extraction and tryptic digestion from esophagogastric tissue (23).

Biopsies between 30 mg and 60 mg in weight were maintained on dry ice until rapid disruption at room temperature (RT) in low-binding micro-centrifuge tubes containing 1 mm ceramic beads (Matrix D, MP Bio, Santa Ana, CA) by rapid shaking in a bench-top homogenizer (FastPrep-24, MP Bio) for 40 s at 6ms^−1^. Homogenates were dissolved in FASP lysis buffer (4% w/v SDS, 100 mm Tris/HCl, 100 mm DTT, pH 7.6), mixed for 20 min at RT and sonicated at maximum amplitude, for 30 s, on ice, using a needle sonicator (Bioruptor, Diagenode, Liège, Belgium). Sonicated lysates were heated for 5 min at 95 °C and clarified by centrifugation at 14,000g for 5 min at 20^p^C before buffer-exchange as per the published FASP protocol (23). Trypsinization was performed off-column, overnight at 37 °C at a ratio of 100:1 (lysate protein mass : trypsin mass) using sequencing grade modified trypsin (Promega, Madison, WI) as per manufacturer's instructions. Protein concentration was determined using a modified Lowry procedure as per the manufacturer's recommendations (RC-DC, Bio-Rad, Hercules, CA).

##### Isobaric labeling and Fractionation of Tryptic peptides

Tryptic peptides from each tissue sample were independently labeled with one of the 6 Tandem Mass Tag (TMT) reagents (Thermo Scientific, San Jose, CA) in technical duplicate per manufacturer's instructions (Fig. 1). Labeled peptides from a single patient (6 reporter ions) were pooled, desalted on a Macro SpinColumn C18 (Harvard Apparatus, Holliston, MA) and separated into 24 fraction by OFFGEL electrophoresis as previously described (24).

##### Liquid Chromatography

Dried, desalted peptide fractions were reconstituted in 5% (v/v) acetonitrile, 0.1% (v/v) Formic Acid (FA) in dH_2_O and ∼0.5 μg loaded onto a homemade, 100 μm internal diameter, 20 mm long trapping column packed with 200 Å, 5 μm Magic C18 AQ (Michrom, Auburn, CA). Trapped peptides were eluted into a 75 μm internal diameter, 150 mm long analytical column packed with 100 Å, 3 μm Magic C18 AQ (Microcom).

For ultraperformance liquid chromatography (UPLC), peptides were separated using a variable solvent gradient created by a combination of 0.1% (v/v) FA in dH_2_O (solvent A) and 0.1% (v/v) FA in acetonitrile (solvent B). The gradient was run as follows: 0–1 mins, 95% (A) and 5% (B), 1–56 mins, 65% (A) and 35% (B), 66–76 mins, 20% (A) and 80% (B) using a flow rate of 220 nL/min.

##### Mass Spectrometry

Peptides were analyzed in positive ion mode after electrospray ionisation on an LTQ-Orbitrap Velos mass spectrometer (Thermo Scientific, San Jose, CA). For MS survey scans, the Orbitrap (OT) resolution was set to 60,000 and the ion population was set to 5 × 10^5^ with an *m*/*z* window from 400 to 2000. A maximum of three precursor ions with the greatest peak intensities were selected for both collision-induced dissociation (CID) and high-energy C-trap dissociation (HCD) in the LTQ with analysis in the OT. For fragment ion analysis in the LTQ, the ion population was set to 7 × 10^3^ (isolation width of 2 m/z) whereas for detection in the OT, the ion population was set to 2 × 10^5^ (isolation width of 2.5 m/z), with resolution of 7500, first mass at *m*/*z* = 100, and maximum injection time of 750 ms. The normalized collision energies were set to 35% for CID and 60% for HCD.

##### Protein Identification

Protein identifications were made using the Easyprot platform (v2.3 build 720, Swiss Institute of Bioinformatics) (25). Data manipulation was performed using Excel (Version 14.0.6129.5000, Microsoft Office Professional 2010), R (version 2.15.1, General Public License), and custom scripts written in Perl (version 5.18.0, General Public License).

Thermo RAW files were converted to peak lists using ReAdW (version 4.3.1, ThermoFinnigan) and CID and HCD spectra were merged for simultaneous identification and quantification as previously described (26). Peaklist files were searched against the Uniprot human reference proteome (release 09/01/2013, containing 87,613 entries) using Phenyx® (version 2.6.1, GeneBio) (27) with a precursor ion tolerance of 10 parts per million and a fragment ion tolerance of 0.6 Da. Variable peptide modifications included TMT-modified N termini and lysines (additional 229.1629 Da) and oxidized methionines, with carbamidomethylation of cysteines set as a fixed modification. Trypsin was selected as the digestion enzyme, with one potential missed cleavage and a minimum of a single-tryptic terminus, a peptide length of 6 amino acids and a z-score of 4 were required.

All data sets were searched separately, once using a forward and once using a reversed protein database. The peptide false discovery rate was set to 1%. A single unique peptide was accepted for protein identification. Identified peptide sequences, scores, precursor *m*/*z*, corresponding proteins, protein coverage and raw data files have been deposited to the ProteomeXchange Consortium (http://proteomecentral.proteomexchange.org) via the PRIDE partner repository (28) with the dataset identifier PXD004962.

##### Protein Quantitation

For relative protein quantitation, the TMT reporter ion intensities were extracted for each peptide. An isotopic purity correction was performed within Easyprot for each reporter based on the isotopic distribution of the sixplex-TMT reporters provided by the manufacturer.

The ratios of peptide expression between EAC and normal esophagus (TvE) and EAC and normal gastric epithelium (TvG) were calculated for each peptide as the ratio of 126/128 reporter ion intensities and 126/130 reporter intensities respectively. This was repeated for 127/129 (TvO) and 127/131 (TvG) reporter ions as a technical replicate. The geometric mean peptide TvE and TvG ratios were calculated for each protein to derive an estimate of the relative protein expression between patient-matched tissue types, and limiting the skew introduced by outlier ratios (29). Because of the ambiguity from potentially shared peptides, all protein isoforms were grouped under the parent protein identifier (Uniprot accession). The variance of each ratio and the number of peptides contributing to the mean were used for subsequent significance calculations.

##### Deriving a Mean Expression Ratio across Replicates

A mean expression value was derived across pooled technical and biological replicates by a meta-analysis approach using a fixed-effect model (30). The inverse of the variance in peptide reporter ratios was used to weight the contribution of protein expression from a replicate to the mean. In this manner, replicates with many peptides detected with similar relative tissue expression (low variance) for a protein contribute a greater proportion to the mean expression value (see supplementary Methods). The unweighted arithmetic mean and variance of the ratios across experiments was calculated for proteins identified by a single unique peptide in multiple replicates. The log_2_-transformed protein expression ratios (TvE and TvG) were median normalized to account for systematic errors during TMT labeling such as minor variations in protein loading.

##### Statistical Tests

The intertissue ratios (TvE and TvG) were compared with technical replicates (TvT, EvE and TvT, GvG) using Welch's modified *t* test (31) as the variances between them differed significantly (32); Fligner-Killeen test *p* < 10^−10^, supplemental Fig. S1. Welch's *t* test was used to test the hypothesis that the relative protein expression between different tissues was not different from relative protein expression between technical replicates with “*n*” defined as the number of peptides contributing to the mean ratio (see supplementary Methods) (33). All *p* values were corrected using the Benjamini-Yekuteili method to control for multiple hypothesis testing (34). Significance was defined as a false discovery rate (FDR)-corrected *p* < 0.05 and *p* values were two-tailed. Relative quantitation and significance are provided for all identified proteins (uploaded supplementary file; All_quantitation.xlsx).

##### Immunohistochemistry

For verification of relative protein expression, 4 μm sections were cut from formalin-fixed, paraffin-embedded (FFPE) blocks derived from the same resection specimens used to collect the fresh tissue for this study and representing normal esophagus, normal stomach and EAC. Sections were subjected to immunohistochemical staining using standard techniques as previously described (35). Staining conditions were optimized for each antibody, included a no primary antibody control for each protein and are detailed in supplemental Table S2.

##### Tissue Microarray

After institutional approval (R&D ID 2006/W/PA/01), FFPE blocks comprising normal esophagus, normal stomach, EAC, normal lymph nodes and, if present, lymph node metastasis were identified for an independent cohort of 115 patients undergoing esophageal resection for EAC between 1994 and 2005 (supplemental Table S1). Representative cores (0.6 mm) were transferred to a separate paraffin block as a tissue microarray (TMA). TMA sections (4 μm) were stained as previously and scored using a modified Allred method (36) by an expert histopathologist. Intensity was graded 0–3 (0 = nil, 1 = weak, 2 = moderate, 3 = strong) and frequency was graded 0–4 (0 = 0%, 1 = 1–10%, 2 = 10 = 50%, 3 = 50–80%, 4 = 80–100%). The sum of intensity + frequency was calculated for each scorable core. Not all cores could be scored because of loss of material during IHC or lack of the appropriate tissue type in the core.

##### Western Blotting and RNA Interference

Lysates from each of the esophageal cell types growing under basal conditions were resolved by SDS-PAGE (20 μg protein per lane, 12% SDS gel) as previously described (37). Western blots were probed with primary antibodies directed against ARHGDIB (ab88317, Abcam, Cambridge, UK, 1/500 overnight at 4 °C) or β-Actin (AC-15, Sigma, Dorset, UK, 1/5000, 2 h at RT) followed by secondary incubation with rabbit anti-mouse or goat anti-rabbit antibodies conjugated to either the IRDye™ 680RD or IRDye™ 800CW ((Li-Cor Biosciences, Lincoln, NE, 1/2000, 2 h at RT protected from light). Blots were imaged using the Odyssey SA system (Li-Cor Biosciences) as per manufacturer's recommendations.

For siRNA experiments, OE33 cells were transfected with vector, nontargeting scrambled sequence siRNA (siGENOME, Non-Targeting siRNA#3, Dharmacon, Lafayette, CO) or siRNA to ARHGDIB (siGENOME, SMARTPool, Dharmacon) as previously described (37). Cells were harvested 72 h after transfection and lysates resolved by Western blotting as previously.

## RESULTS

A total of 6349 proteins were identified and quantified across all samples corresponding to 4772 unique Entrez GeneIDs with 744 proteins quantified in both replicates from all seven samples. The protein identifications per patient are shown in Table III.

**Table III:** Protein identifications by patient

Patient	Total proteins (1% peptide FDR, 1 unique peptide per protein)	Unique to patient
Pt44	2901	368
Pt46	2534	256
Pt48	3309	550
Pt51	3327	503
Pt53	2904	280
Pt60	2369	220
Pt61	2828	264

### 

#### 

##### Reproducibility of Quantitation

**Fig. 2.**
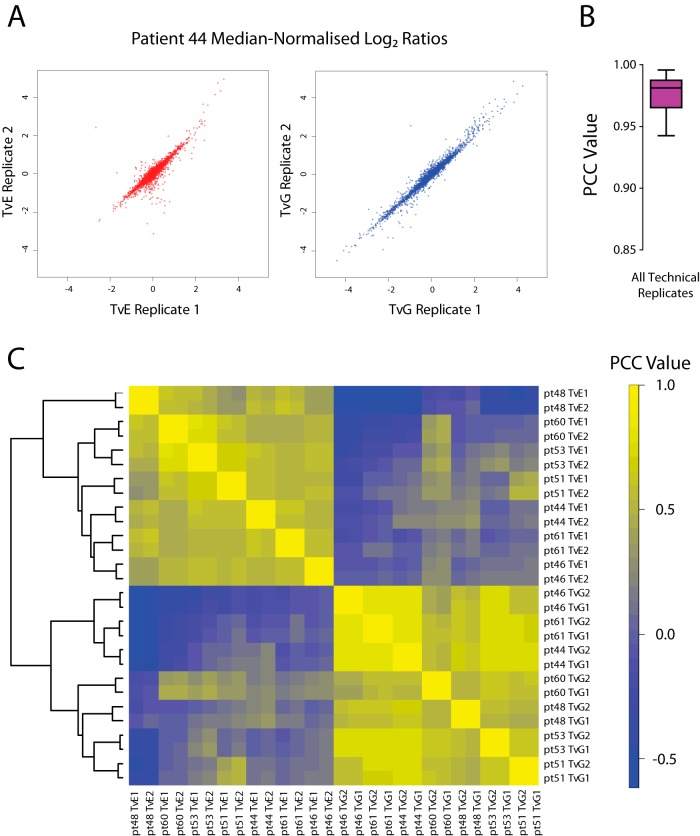
**Correlation of technical replicates.** *A*, Median-normalized log_2_ ratios are displayed for two technical replicates of samples from patient 44. TvE = log_2_ ratio of expression between EAC and normal esophagus. TvG = log_2_ ratio of expression between EAC and normal gastric tissue. *B*, Box and whisker plot summarizing the median (line within the box), 25th and 75th percentiles (box limits) and 5th and 95th percentiles (whiskers) for the Pearson's correlation coefficients (PCC) for technical replicates across all experiments (*n* = 14). *C*, Heatmap representation of PCC between technical replicates across all experiments. Unsupervised hierarchical clustering was performed using an agglomerative complete linkage method to generate the similarity dendrogram (left).

The reproducibility of quantification of protein expression was assessed by comparing technical replicates. Expression levels were highly correlated between technical replicates from the same patient's tissues (Fig. 2*A*, 2*B*, median Pearson correlation coefficient (PCC) = 0.9811, *p* < 0.001). There was also a reassuringly good correlation between different patients (biological replicates) when TvE ratios were considered (Fig. 2*C*, median PCC = 0.555, *p* < 0.001) demonstrating concordance of the protein expression from histologically similar tissues within a relatively clinically homogeneous patient cohort. As expected, there was no significant correlation between TvE and TvG ratios across patients (Fig. 2*C*, median PCC = 0.0115, *p* > 0.05) underscoring the diversity in protein expression of the tissues studied.

##### A Map of Protein Expression across Esophagogastric Tissues

Protein expression was quantified by the ratio of TMT reporter ion intensities. Ratios were not, however, calculable for proteins exclusively expressed in one tissue and these proteins may have been excluded from our analysis despite their biological importance. Several peptides were manually identified with no reporter ion expression from one or two tissue types. In the context of other unique peptides identified from the same protein, however, no proteins with entirely tissue-specific expression could be identified in this study.

**Fig. 3.**
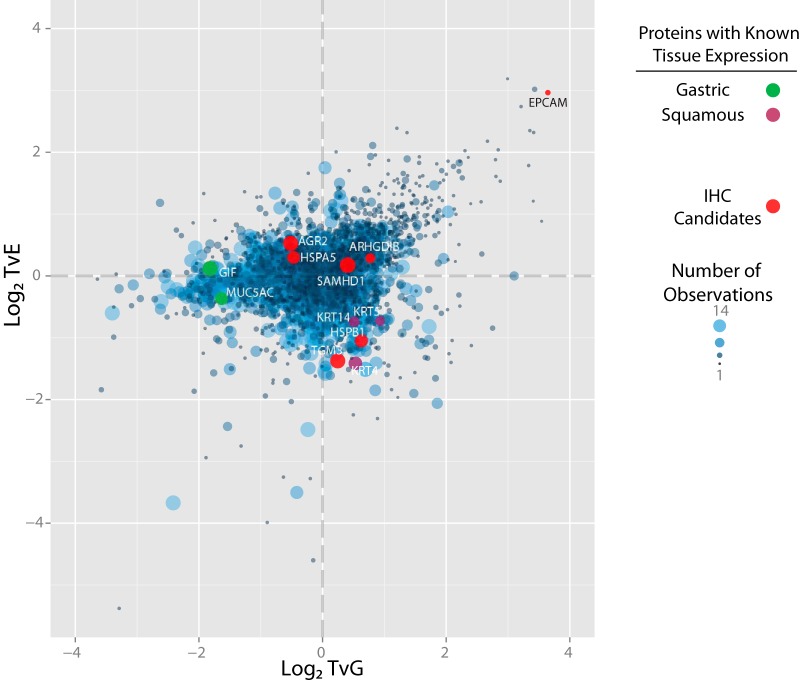
**Distribution of Significantly Dysregulated Proteins.** Significance was defined as an FDR-corrected *p* < 0.05 for the comparison of TvE and the mean of TvT and EvE ratios or for the comparison of TvG and the mean of TvT and GvG ratios. Each point on this figure represents a single protein that is significantly dysregulated in at least one tissue type. Proteins have been plotted according to their log_2_ expression ratios and points sized per the frequency of observation by MS across pooled biological and technical replicates (range 1–14). Selected proteins, labeled by their official gene names, are highlighted in green for proteins known to be specifically expressed in gastric tissue, purple for proteins known to be expressed in squamous tissue and red for proteins selected for further validation by IHC.

For the 4181 proteins identified by more than one peptide in more than one replicate, and the 2154 proteins identified by more than one peptide in a single replicate or a single peptide in more than one replicate a mean expression value and variance were derived (uploaded supplementary file; All_quantitation.xlsx). Those 14 proteins identified by a single peptide and only in a single replicate were considered low confidence identifications and were excluded from further analysis. The expression ratios for the 3082 proteins significantly dysregulated between tissues (FDR-corrected *p* < 0.05 for either TvG or TvE ratios) were used to produce a two-dimensional protein expression map with vectors of TvG expression on the *x* axis and TvE on the *y* axis (Fig. 3).

Proteins overexpressed in EAC would be expected to have both high TvE and TvG ratios and therefore be identified in the upper right quadrant of the protein expression plot, discrete from other nonspecifically expressed proteins. It was proposed that other proteins with tissue-specific expression patterns would also be closely associated on the plot. To test this hypothesis, proteins with an established expression pattern were considered. Gastric Intrinsic Factor (GIF) and Mucin 5AC are known to be specifically highly expressed in gastric epithelium (38, 39). Reassuringly, both proteins were closely associated on the protein expression map and demonstrated similar expression in EAC and normal esophageal tissue but high expression in gastric epithelium (Fig. 3). Similarly, Keratins 4, 5, and 14 are known to be highly expressed in esophageal squamous epithelium (40, 41) and were all clustered together. It was possible that the tissue expression pattern of other proteins could be inferred from their location on this map.

To test this, a further two proteins predicted to be upregulated in squamous tissue (heat shock protein family B (small) member 1; HSPB1 and transglutaminase 3; TGM3), two proteins upregulated in tumor but also generally expressed (SAM and HD domain containing deoxynucleoside triphosphate triphosphohydrolase 1; SAMHD1 and Rho GDP dissociation inhibitor beta; ARHGDIB), two proteins upregulated in both tumor and gastric tissue (anterior gradient 2, protein disulfide isomerase family member; AGR2 and heat shock protein family A (Hsp70) member 5; HSPA5) and one protein predicted to be specifically highly expressed in tumor (epithelial cell adhesion molecule; EPCAM) (supplemental Table S3) were selected for verification by immunohistochemistry (IHC).

##### Verification of Protein-expression Differences Across EAC and Patient-matched Normal Tissues

**Fig. 4.**
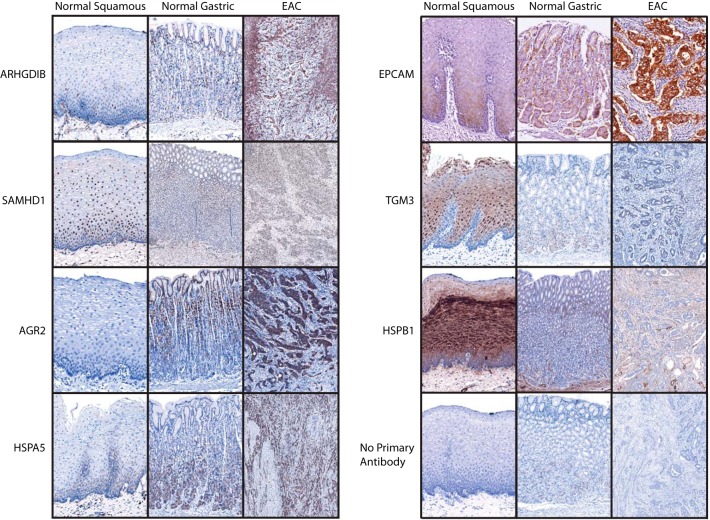
**IHC staining pattern for selected proteins.** Representative images are provided highlighting the staining pattern from EAC and matched normal tissues from the original resection specimens used to derive the fresh frozen biopsies.

Sections from the FFPE tissue blocks from the original resection specimens used to derive the fresh tissue samples for proteomic analysis were subjected to IHC (Fig. 4). All five of the proteins predicted to be upregulated in EAC compared with normal esophagus (SAMHD1, ARHGDIB, AGR2, HSPA5, EPCAM) showed higher expression in the tumor sections compared with squamous epithelium. Similarly, the proteins predicted to be expressed preferentially in squamous epithelium; HSPB1 and TGM3, showed the highest expression in normal squamous esophageal tissue (supplemental Table S3).

Although false positive homogeneous cytoplasmic staining was observed in all or scattered basal crypt epithelial cells in most gastric tissue sections (Fig. 4; No primary antibody) (42), both HSPA5 and AGR2 demonstrated true gastric epithelial staining as expected and were overexpressed in EAC compared with squamous epithelium.

SAMHD1 showed more widespread nuclear expression across tissues but was mildly upregulated in EAC cells, as predicted. In contrast, EPCAM was very highly expressed in tumor cells with only moderate staining in basal epithelial squamous cells and gastric epithelial cells. Although overall expression of ARHGDIB was indeed higher in EAC compared with normal squamous and gastric tissue, this staining was observed in stromal cells, most likely lymphocytes, rather than epithelial-derived tumor cells.

These findings support the accuracy of the quantitative proteomic approach for each of the seven candidates selected. As expected from the proteomic data, EPCAM demonstrated the greatest specificity for tumor cells.

**Fig. 5.**
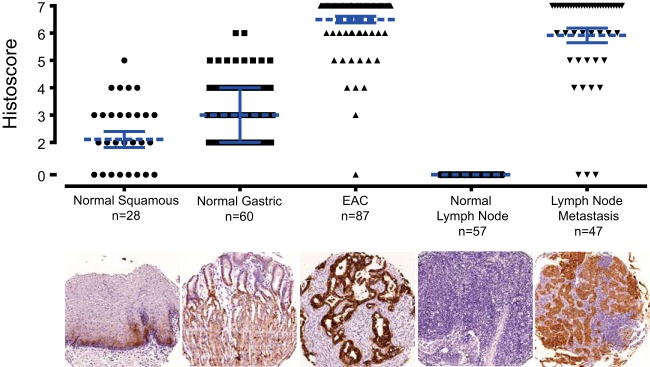
**EPCAM expression in esophageal adenocarcinoma, lymph node metastases and corresponding normal tissues.** Raw histoscores are presented as points with medians as dashed blue lines. Error bars in blue represent interquartile ranges. The number of scorable cores examined is presented below each column as well as a representative core image.

To determine the specificity of EPCAM for EAC cells compared with surrounding normal tissues, protein expression was determined by IHC using a tissue microarray consisting; normal gastric tissue, normal squamous tissue, uninvolved lymph nodes, involved lymph nodes and primary tumor samples from resection specimens from 115 patients whom had undergone surgical resection for EAC (Fig. 5). EPCAM was expressed at low levels in basal squamous epithelial cells and low to moderate levels in gastric epithelium. In contrast EPCAM was highly expressed in EAC and was expressed at higher levels than the median normal gastric or normal esophageal epithelial expression in 98% of tumors. This high specificity was demonstrated in metastatic lesions as well as primary tumors with no EPCAM expression detectable in normal lymph nodes but high expression in 93% of lymph node metastases.

**Fig. 6.**
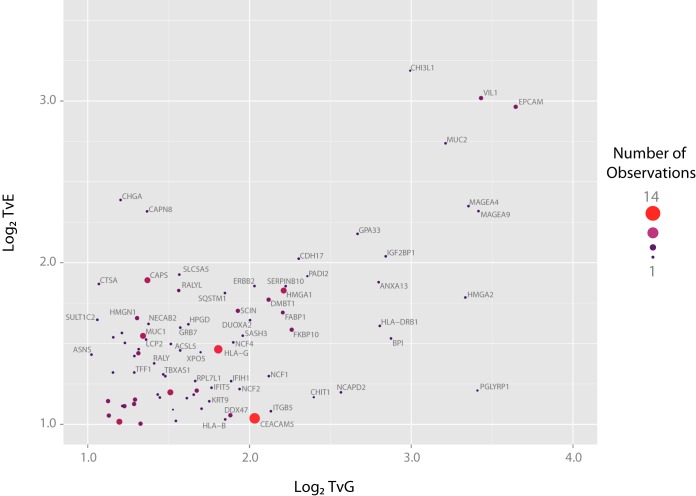
**Annotation of Proteins Highly Expressed in Tumors Compared with Normal Esophageal and Gastric Epithelium.** Points are identical to Fig. 3 but only proteins within a selected expression range are shown. Proteins have been annotated with their official gene names and are sized according to the frequency of observation by MS across pooled technical and biological replicates (range 1–14).

This proteomic strategy has therefore demonstrated the ability to detect relatively specific markers of EAC. Those proteins predicted to be highly expressed in EAC compared with surrounding normal tissues, including several novel candidate therapeutic targets, are presented in more detail in Fig. 6.

## DISCUSSION

There is a need to identify selective markers of esophageal adenocarcinoma for early diagnosis, to enhance clinical staging and direct novel therapies. This shotgun proteomic study compared protein expression from matched esophageal adenocarcinoma, normal esophagus and normal gastric samples from seven patients and provides quantitative data on protein expression for over 6000 proteins across these tissues. A comparative analysis approach was employed to select tumor-specific proteins and this method was verified to be accurate by IHC. Multiple novel tumor-specific proteins are proposed and EPCAM was demonstrated to be specifically overexpressed in primary tumors and lymph node metastases.

Incomplete proteome coverage remains a significant limitation of all proteomic studies, however, and only 744 proteins (12%) were detected in all technical replicates across the seven patients' tissues. Despite this intrinsic limitation of proteomic studies, the technical reproducibility of protein quantitation was very high and a strategy was developed to identify significantly dysregulated proteins.

The combination of TvE and TvG ratios for each quantified protein allowed the generation of a 2D expression map. The relative quantitative accuracy of this approach was confirmed first by evaluation of proteins with a well validated expression profile (GIF, Muc5AC, Keratin 4,5,14) and then subsequently by IHC for 7 further proteins with varied expression profiles (ARHGDIB, SAMHD1, AGR2, HSPA5, EPCAM, TGM3, HSPB1). In each case the observed staining pattern mirrored the expected expression from the proteomic data (supplemental Table S3). This provides confidence in the predicted expression for proteins with an unknown pattern.

Both TGM3 and HSPB1 were found to be expressed at high levels in squamous epithelium compared with EAC, as expected from our proteomic data and as reported in previous work (43). In contrast, AGR2 was found to be expressed in gastric epithelium and EAC with no expression observed in squamous epithelia. Similarly, HSPA5 was found to be expressed in both EAC and gastric epithelium. These findings agree with previous reports (44–48). HSPA5 is thought to play a key role in the regulation of the unfolded-protein response and this expression pattern may reflect an increased protein chaperone demand in secretory cells (48, 49). Indeed, AGR2 has a function in protein homeostasis and secretion (50).

SAMHD1 exhibited nuclear expression in both epithelial and nonepithelial cells with the highest expression in EAC. It has a reported role in restricting HIV replication and modulating the immune response in T cells (51) but there is limited data for its role in cancer and this would be worthy of further study.

ARHGDIB was overexpressed in EAC sections compared with normal squamous and normal gastric tissue. The ARHGDIB positive cells appeared, however, to be lymphocytes rather than epithelial-derived tumor cells. This expression pattern has been observed for ARHGDIB with a different antibody (47) and a multitissue study suggested expression was restricted to hematopoietic cells (52).

Divergent roles for ARHGDIB have subsequently been proposed in the literature with some evidence for a role in the suppression of metastasis in bladder cancer (53) and in contrast a proinvasive role in gastric cancer (54). One previous proteomic study reported ARHGDIB overexpression in EAC compared with Barrett's epithelium at both the mRNA and protein level (22). In contrast to our study, the previous work presented cytoplasmic and membrane staining in epithelial cells with minimal stromal positivity in EAC sections. The specificity of the rabbit anti-ARHGDIB antibody used in that study was not demonstrated in the manuscript. In contrast the antibody in this study identifies a protein of the predicted mass (48 kDA) in a panel of esophageal cell lines by Western blotting and is specific for that protein as confirmed by siRNA (supplemental Fig. S2). The variable expression of ARHGDIB noted in the panel of esophageal cell lines may reflect context-dependent regulation. Staining across a larger number of esophageal tumors could establish greater confidence over the cell-specific expression profile.

The cancer antigen EPCAM was predicted to be highly expressed in EAC cells compared with surrounding normal tissues and this was indeed observed. This has previously been demonstrated for several cancer types, including esophageal (55, 56). EPCAM is a cell adhesion molecule that is highly expressed on the cell membrane and may possess a signaling role through the regulation of cell proliferation via a cleaved intracellular domain (57). Because of its high specificity for malignancy, EPCAM-based assays have been developed to detect circulating tumor cells although these are less sensitive for mesenchymal tumors (58).

The expression of EPCAM was examined in detail by IHC in an independent cohort of 115 esophageal adenocarcinomas with matched normal and metastatic tissues. EPCAM was highly expressed in the clear majority of EACs with higher EPCAM histoscores observed in 85/87 EACs than the median gastric or esophageal scores. EPCAM was also highly specifically expressed in lymph node metastases compared with surrounding normal lymph nodes raising the possibility that it could exploited to enhance clinical staging using novel techniques (59).

These data agree with previous work demonstrating overexpression of EPCAM in EAC compared with surrounding normal tissues (56). A further study identified disseminated tumor cells from bone marrow and lymph nodes in patients with esophageal cancer and, although the primary tumors predominantly expressed high levels of EPCAM, EPCAM expression was only observed in 37% of disseminated tumor cells from bone marrow aspirates (60). A reduction in EPCAM expression by RNA interference increased migration *in vitro* and the authors proposed that EPCAM expression reduced during the process of invasion as cells adopted a more mesenchymal phenotype. This may have clinical implications if anti-EPCAM therapies are to be considered.

Samples were obtained from six men and one woman (Table II) reflecting the 4-fold greater prevalence of EAC in men (18). Although this bias could limit the applicability of this study to women with EAC, no differences were noted in the expression patterns of EPCAM across tissue types from men (*n* = 97) and women (*n* = 18) in the TMA.

EAC exhibits a high frequency of DNA mutation and mutant proteins are highly likely to be tumor specific (12). A limitation of this study is that mass spectra were searched against a protein database containing only wildtype proteins, however, so that mutant proteins could not be identified. An alternative strategy is to generate mutant protein databases using tumor genome sequencing data, either specific to the patient's tumor or using commonly identified variants and search mass spectra against these (61). This proteogenomic approach has the potential to reveal tumor-specific proteins, however, significant technical challenges remain in controlling the protein database size and false-discovery rate (62).

Potentially because of this limitation, no entirely tumor-specific proteins could be identified in this study. Importantly, however, a group of proteins highly expressed in tumors relative to surrounding normal tissues was proposed (Fig. 6). Immunotherapeutic trials are already underway in other cancer types with agents directed against several of these including EPCAM (63), glycoprotein A33; GPA33 (64, 65), mucin 1, cell surface associated; MUC1 (66) and melanoma antigen; MAGE (67) proteins. If the expression of these can be validated to be specific to EAC cells over surrounding tissues, there would be a compelling rationale to expand trials to include patients with EAC and to develop specific imaging tools to these targets to enhance clinical staging.

These data have been presented, in part, at the Human Proteome Organisation (HUPO) World Congress in 2012 and the Association of Upper-GI Surgeons of Great Britain and Ireland (AUGIS) in 2013 and published in abstract form (British Journal of Surgery 2013;100(S8):55).

## DATA AVAILABILITY

Corresponding proteins, protein coverage and raw data files have been deposited to the ProteomeXchange Consortium (http://proteomecentral.proteomexchange.org) via the PRIDE partner repository (28) with the dataset identifier PXD004962. Relative quantitation and significance are provided for all identified proteins (uploaded supplementary file; All_quantitation.xlsx).

## Supplementary Material

Supplemental Data

## Footnotes

^1^ The abbreviations used are:

EAC: Esophageal adenocarcinoma
EGJ: Esophagogastric junction
CT: computed tomography
PET: positron emission tomography
EUS: endoscopic ultrasound
TMT: Tandem Mass Tag
IHC: immunohistochemistry
RT: room temperature
FASP: filter-aided sample preparation
FA: formic acid
dH_2_O: distilled water
NMWCO: nominal molecular weight cut-off
UPLC: ultra-performance liquid chromatography
TvE: ratio of EAC to normal oesophagus expression
TvG: ratio of EAC to normal gastric expression
ESCC: Esophageal squamous cell carcinoma
LVI: lymphovascular invasion
PNI: perineural invasion.

## References

[1] Jemal A., Bray F., Center M. M., Ferlay J., Ward E., and Forman D. (2011) Global cancer statistics. CA Cancer J. Clin. 61, 69–90 21296855 10.3322/caac.20107

[2] Lepage C., Rachet B., Jooste V., Faivre J., and Coleman M. P. (2008) Continuing rapid increase in esophageal adenocarcinoma in England and Wales. Am. J. Gastroenterol. 103, 2694–2699 18853967 10.1111/j.1572-0241.2008.02191.x

[3] Pohl H., and Welch H. (2005) The role of overdiagnosis and reclassification in the marked increase of esophageal adenocarcinoma incidence. J. Natl. Cancer Inst. 97, 142–146 15657344 10.1093/jnci/dji024

[4] Groene O., Cromwell D., Hardwick R., Riley S., Crosby T., and Greeenaway K. (2012) National Oesophago-Gastric Cancer Audit. Royal College of Surgeons of England

[5] Allum W. H., Blazeby J. M., Griffin S. M., Cunningham D., Jankowski J. A., and Wong R., Association of Upper Gastrointestinal Surgeons of Great Britain and Ireland, the British Society of Gastroenterology and the British Association of Surgical Oncology. (2011) Guidelines for the management of oesophageal and gastric cancer. Gut 60, 1449–1472 21705456 10.1136/gut.2010.228254

[6] O'Neill J. R., Kennedy E. D., Save V., Langdale-Brown B., Wall L., Skipworth R. J. E., and Paterson-Brown S. (2017) Patients unfit for neoadjuvant therapy may still undergo resection of locally advanced esophageal or esophagogastric junctional cancer with acceptable oncological results. IJS Oncol. 2, e09 10.1097/IJ9.0000000000000009 PMC5673116 29177210

[7] Dikken J. L., van Sandick J. W., Allum W. H., Johansson J., Jensen L. S., Putter H., Coupland V. H., Wouters M. W., Lemmens V. E., van de Velde C. J., van der Geest L. G., Larsson H. J., Cats A., and Verheij M. (2013) Differences in outcomes of oesophageal and gastric cancer surgery across Europe. Br. J. Surg. 100, 83–94 23180474 10.1002/bjs.8966

[8] Fischer C., Lingsma H., Hardwick R., Cromwell D. A., Steyerberg E., and Groene O. (2016) Risk adjustment models for short-term outcomes after surgical resection for oesophagogastric cancer. Br. J. Surg. 103, 105–116 26607783 10.1002/bjs.9968

[9] McElroy M., Kaushal S., Luiken G. A., Talamini M. A., Moossa A. R., Hoffman R. M., and Bouvet M. (2008) Imaging of primary and metastatic pancreatic cancer using a fluorophore-conjugated anti-CA19–9 antibody for surgical navigation. World J. Surg. 32, 1057–1066 18264829 10.1007/s00268-007-9452-1 PMC4378829

[10] Souza R. F., Krishnan K., and Spechler S. J. (2008) Acid, bile, and CDX: the ABCs of making Barrett's metaplasia. Am. J. Physiol. Gastrointest. Liver Physiol. 295, 1–218 10.1152/ajpgi.90250.2008 18556417

[11] Gertler R., Stein H. J., Langer R., Nettelmann M., Schuster T., Hoefler H., Siewert J. R., and Feith M. (2011) Long-term outcome of 2920 patients with cancers of the esophagus and esophagogastric junction: evaluation of the New Union Internationale Contre le Cancer/American Joint Cancer Committee staging system. Ann. Surg. 253, 689–698 21475008 10.1097/SLA.0b013e31821111b5

[12] Weaver J. M., Ross-Innes C. S., Shannon N., Lynch A., Forshew T., Barbera M., Murtaza M., Ong C. A., Lao-Sirieix P., Dunning M. J., Smith L., Smith M. L., Anderson C. L., Carvalho B., O'Donovan M., Underwood T. J., May A. P., Grehan N., Hardwick R., Davies J., Oloumi A., Aparicio S., Caldas C., Eldridge M. D., Edwards P. A., Rosenfeld N., Tavaré S., Fitzgerald R. C., and OCCAMS Consortium (2014) Ordering of mutations in preinvasive disease stages of esophageal carcinogenesis. Nat. Genet. 46, 837–843 24952744 10.1038/ng.3013 PMC4116294

[13] McGranahan N., Furness A. J., Rosenthal R., Ramskov S., Lyngaa R., Saini S. K., Jamal-Hanjani M., Wilson G. A., Birkbak N. J., Hiley C. T., Watkins T. B., Shafi S., Murugaesu N., Mitter R., Akarca A. U., Linares J., Marafioti T., Henry J. Y., Van Allen E. M., Miao D., Schilling B., Schadendorf D., Garraway L. A., Makarov V., Rizvi N. A., Snyder A., Hellmann M. D., Merghoub T., Wolchok J. D., Shukla S. A., Wu C. J., Peggs K. S., Chan T. A., Hadrup S. R., Quezada S. A., and Swanton C. (2016) Clonal neoantigens elicit T cell immunoreactivity and sensitivity to immune checkpoint blockade. Science 351, 1463–1469 26940869 10.1126/science.aaf1490 PMC4984254

[14] Rosenberg S. A., Yang J. C., Sherry R. M., Kammula U. S., Hughes M. S., Phan G. Q., Citrin D. E., Restifo N. P., Robbins P. F., Wunderlich J. R., Morton K. E., Laurencot C. M., Steinberg S. M., White D. E., and Dudley M. E. (2011) Durable complete responses in heavily pretreated patients with metastatic melanoma using T-cell transfer immunotherapy. Clin. Cancer Res. 17, 4550–4557 21498393 10.1158/1078-0432.CCR-11-0116 PMC3131487

[15] Singhal R., Carrigan J. B., Wei W., Taniere P., Hejmadi R. K., Forde C., Ludwig C., Bunch J., Griffiths R. L., Johnson P. J., Tucker O., Alderson D., Günther U. L., and Ward D. G. (2013) MALDI profiles of proteins and lipids for the rapid characterisation of upper GI-tract cancers. J. Proteomics. 80, 207–215 23376328 10.1016/j.jprot.2013.01.016

[16] Geiger T., Velic A., Macek B., Lundberg E., Kampf C., Nagaraj N., Uhlen M., Cox J., and Mann M. (2013) Initial quantitative proteomic map of 28 mouse tissues using the SILAC mouse. Mol. Cell. Proteomics. 12, 1709–1722 23436904 10.1074/mcp.M112.024919 PMC3675825

[17] Kim M. S., Pinto S. M., Getnet D., Nirujogi R. S., Manda S. S., Chaerkady R., Madugundu A. K., Kelkar D. S., Isserlin R., Jain S., Thomas J. K., Muthusamy B., Leal-Rojas P., Kumar P., Sahasrabuddhe N. A., Balakrishnan L., Advani J., George B., Renuse S., Selvan L. D., Patil A. H., Nanjappa V., Radhakrishnan A., Prasad S., Subbannayya T., Raju R., Kumar M., Sreenivasamurthy S. K., Marimuthu A., Sathe G. J., Chavan S., Datta K. K., Subbannayya Y., Sahu A., Yelamanchi S. D., Jayaram S., Rajagopalan P., Sharma J., Murthy K. R., Syed N., Goel R., Khan A. A., Ahmad S., Dey G., Mudgal K., Chatterjee A., Huang T. C., Zhong J., Wu X., Shaw P. G., Freed D., Zahari M. S., Mukherjee K. K., Shankar S., Mahadevan A., Lam H., Mitchell C. J., Shankar S. K., Satishchandra P., Schroeder J. T., Sirdeshmukh R., Maitra A., Leach S. D., Drake C. G., Halushka M. K., Prasad T. S., Hruban R. H., Kerr C. L., Bader G. D., Iacobuzio-Donahue C. A., Gowda H., and Pandey A. (2014) A draft map of the human proteome. Nature. 509, 575–581 24870542 10.1038/nature13302 PMC4403737

[18] O'Neill J. R., Stephens N. A., Save V., Kamel H. M., Phillips H. A., Driscoll P. J., and Paterson-Brown S. (2013) Defining a positive circumferential resection margin in oesophageal cancer and its implications for adjuvant treatment. Br. J. Surg. 100, 1055–1063 23616367 10.1002/bjs.9145

[19] Cai Z., Zhao J. S., Li J. J., Peng D. N., Wang X. Y., Chen T. L., Qiu Y. P., Chen P. P., Li W. J., Xu L. Y., Li E. M., Tam J. P., Qi R. Z., Jia W., and Xie D. (2010)A combined proteomics and metabolomics profiling of gastric cardia cancer reveals characteristic dysregulations in glucose metabolism. Mol. Cell. Proteomics. 9, 2617–2628. 20699381 10.1074/mcp.M110.000661 PMC3101851

[20] Besson D., Pavageau A.H., Valo I., Bourreau A., Bélanger A., Eymerit-Morin C., Moulière A., Chassevent A., Boisdron-Celle M., Morel A., Solassol J., Campone M., Gamelin E., Barré B., Coqueret O., and Guette C. (2011) A quantitative proteomic approach of the different stages of colorectal cancer establishes OLFM4 as a new nonmetastatic tumor marker. Mol. Cell. Proteomics 10, M111.009712 10.1074/mcp.M111.009712 PMC3237075 21986994

[21] Peng D., Sheta E. A., Powell S. M., Moskaluk C. A., Washington K., Goldknopf I. L., and El-Rifai W. (2008) Alterations in Barrett's-related adenocarcinomas: A proteomic approach. Int. J. Cancer 122, 1303–1310 18000824 10.1002/ijc.23258

[22] Zhao J., Chang A. C., Li C., Shedden K. A., Thomas D. G., Misek D. E., Manoharan A. P., Giordano T. J., Beer D. G., and Lubman D. M. (2007) Comparative proteomics analysis of Barrett metaplasia and esophageal adenocarcinoma using two-dimensional liquid mass mapping. Mol. Cell. Proteomics 6, 987–999 16829691 10.1074/mcp.M600175-MCP200

[23] Wiœniewski J.R., Zougman A., Nagaraj N., and Mann M. (2009) Universal sample preparation method for proteome analysis. Nat. Methods 6, 359–362 19377485 10.1038/nmeth.1322

[24] Dayon L., Turck N., Kienle S., Schulz-Knappe P., Hochstrasser D. F., Scherl A., and Sanchez J. C. (2010) Isobaric tagging-based selection and quantitation of cerebrospinal fluid tryptic peptides with reporter calibration curves. Anal. Chem. 82, 848–858 20058875 10.1021/ac901854k

[25] Gluck F., Hoogland C., Antinori P., Robin X., Nikitin F., Zufferey A., Pasquarello C., Fétaud V., Dayon L., Müller M., Lisacek F., Geiser L., Hochstrasser D., Sanchez J.C., and Scherl A. (2013) EasyProt–an easy-to-use graphical platform for proteomics data analysis. J. Proteomics 79, 146–160 23277275 10.1016/j.jprot.2012.12.012

[26] Dayon L., Pasquarello C., Hoogland C., Sanchez J. C., and Scherl A. (2010) Combining low- and high-energy tandem mass spectra for optimized peptide quantification with isobaric tags. J. Proteomics 73, 769–777 19903544 10.1016/j.jprot.2009.10.015

[27] Colinge J., Masselot A., Giron M., Dessingy T., and Magnin J. (2003) OLAV: towards high-throughput tandem mass spectrometry data identification. Proteomics 3, 1454–1463 12923771 10.1002/pmic.200300485

[28] Vizcaíno J. A., Côté R.G., Csordas A., Dianes J.A., Fabregat A., Foster J.M., Griss J., Alpi E., Birim M., Contell J., O'Kelly. G, Schoenegger A., Ovelleiro D., Pérez-Riverol Y., Reisinger F., Ríos D., Wang R., and Hermjakob H. (2013) The PRoteomics IDEntifications (PRIDE) database and associated tools: status in 2013. Nucleic Acids Res. 41, D1063–D1069 23203882 10.1093/nar/gks1262 PMC3531176

[29] Shaffer J. (1992) Caution on the use of variance ratio: A comment. Rev. Educational Res. 62, 429–432

[30] Borenstein M. (2009) Introduction to meta-analysis. Chichester, U.K.: John Wiley & Sons

[31] Ruxton G. (2006) The unequal variance t-test is an underused alternative to Student's t-test and the Mann-Whitney U test. Behavioural Ecol. 17, 688–690

[32] Conover W., Johnson M., and Johnson M. (1981) A comparative study of tests for homogeneity of variances, with applications to the outer continental shelf bidding data. Technometrics 23, 351–361

[33] Welch B. (1947) The generalization of “Student's” problem when several different population variances are involved. Biometrika. 34, 28–35 20287819 10.1093/biomet/34.1-2.28

[34] Benjamini Y., and Yekutieli D. (2001) The control of the false discovery rate in multiple testing under dependency. Ann. Statistics 29, 1165–1185

[35] Murray E., Hernychová L., Scigelova M., Ho J., Nekulova M., O'Neill J. R., Nenutil R., Vesely K., Dundas S. R., Dhaliwal C., Henderson H., Hayward R. L., Salter D. M., Vojtìšek B., and Hupp T. R. (2014) Quantitative proteomic profiling of pleomorphic human sarcoma identifies CLIC1 as a dominant pro-oncogenic receptor expressed in diverse sarcoma types. J. Proteome Res. 13, 2543–2559 24661138 10.1021/pr4010713

[36] Allred D. C., Harvey J. M., Berardo M., and Clark G. M. (1998) Prognostic and predictive factors in breast cancer by immunohistochemical analysis. Mod. Pathol. 11, 155–168 9504686

[37] Huart A. S., MacLaine N. J., Meek D. W., and Hupp T. R. (2009) CK1alpha plays a central role in mediating MDM2 control of p53 and E2F-1 protein stability. J. Biol. Chem. 284, 32384–32394 19759023 10.1074/jbc.M109.052647 PMC2781653

[38] Freeman T. C., Ivens A., Baillie J. K., Beraldi D., Barnett M. W., Dorward D., Downing A., Fairbairn L., Kapetanovic R., Raza S., Tomoiu A., Alberio R., Wu C., Su A. I., Summers K. M., Tuggle C. K., Archibald A. L., and Hume D. A. (2012) A gene expression atlas of the domestic pig. BMC Biol. 10, 90 23153189 10.1186/1741-7007-10-90 PMC3814290

[39] Goebel M., Stengel A., Lambrecht N. W., and Sachs G. (2011) Selective gene expression by rat gastric corpus epithelium. Physiol. Genomics 43, 237–254 21177383 10.1152/physiolgenomics.00193.2010 PMC3068518

[40] Takashima M., Kawachi H., Yamaguchi T., Nakajima Y., Kitagaki K., Sekine M., Iida T., Takemura K., Kawano T., and Eishi Y. (2012) Reduced expression of cytokeratin 4 and 13 is a valuable marker for histologic grading of esophageal squamous intraepithelial neoplasia. J. Med. Dent. Sci. 59, 17–28 23896961

[41] Wang X., Ouyang H., Yamamoto Y., Kumar P.A., Wei T.S., Dagher R., Vincent M., Lu X., Bellizzi A.M., Ho K.Y., Crum C.P., Xian W., and McKeon F. (2011) Residual embryonic cells as precursors of a Barrett's-like metaplasia. Cell 145, 1023–1035 21703447 10.1016/j.cell.2011.05.026 PMC3125107

[42] Ramos-Vara J. A. (2011) Principles and methods of immunohistochemistry. Methods Mol. Biol. 691, 83–96 20972748 10.1007/978-1-60761-849-2_5

[43] Soldes O. S., Kuick R. D., Thompson I. A., Hughes S. J., Orringer M. B., Iannettoni M. D., Hanash S. M., and Beer D. G. (1999) Differential expression of Hsp27 in normal oesophagus, Barrett's metaplasia and oesophageal adenocarcinomas. Br. J. Cancer 79, 595–603 10027336 10.1038/sj.bjc.6690094 PMC2362445

[44] Pohler E., Craig A. L., Cotton J., Lawrie L., Dillon J. F., Ross P., Kernohan N., and Hupp T. R. (2004) Mol. Cell. Proteomics 3, 534–547 14967811 10.1074/mcp.M300089-MCP200

[45] Pizzi M, Fassan M, Realdon S, Balistreri M, Battaglia G, Giacometti C, Zaninotto G, Zagonel V, De Boni M, and Rugge M. (2012) Anterior gradient 2 profiling in Barrett columnar epithelia and adenocarcinoma. Hum. Pathol. 43, 1839–1844 22521076 10.1016/j.humpath.2012.01.004

[46] Thompson D. A., and Weigel R. J. (1998) hAG-2, the human homologue of the Xenopus laevis cement gland gene XAG-2, is coexpressed with estrogen receptor in breast cancer cell lines. Biochem. Biophys. Res. Commun. 251, 111–116 9790916 10.1006/bbrc.1998.9440

[47] Uhlén M., Björling E., Agaton C., Szigyarto C. A., Amini B., Andersen E., Andersson A. C., Angelidou P., Asplund A., Asplund C., Berglund L., Bergström K., Brumer H., Cerjan D., Ekström M., Elobeid A., Eriksson C., Fagerberg L., Falk R., Fall J., Forsberg M., Björklund M. G., Gumbel K., Halimi A., Hallin I., Hamsten C., Hansson M., Hedhammar M., Hercules G., Kampf C., Larsson K., Lindskog M., Lodewyckx W., Lund J., Lundeberg J., Magnusson K., Malm E., Nilsson P., Odling J., Oksvold P., Olsson I., Oster E., Ottosson J., Paavilainen L., Persson A., Rimini R., Rockberg J., Runeson M., Sivertsson A., Sköllermo A., Steen J., Stenvall M., Sterky F., Strömberg S., Sundberg M., Tegel H., Tourle S., Wahlund E., Waldén A., Wan J., Wernérus H., Westberg J., Wester K., Wrethagen U., Xu L. L., Hober S., and Pontén F. (2005) A human protein atlas for normal and cancer tissues based on antibody proteomics. Mol. Cell. Proteomics 4, 1920–1932 16127175 10.1074/mcp.M500279-MCP200

[48] Langer R., Feith M., Siewert J. R., Wester H. J., and Hoefler H. (2008) Expression and clinical significance of glucose regulated proteins GRP78 (BiP) and GRP94 (GP96) in human adenocarcinomas of the esophagus. BMC Cancer 8, 70 18331622 10.1186/1471-2407-8-70 PMC2270853

[49] Hetz C. (2012) The unfolded protein response: controlling cell fate decisions under ER stress and beyond. Nat. Rev. Mol. Cell Biol. 13, 89–102 22251901 10.1038/nrm3270

[50] Chevet E., Fessart D., Delom F., Mulot A., Vojtesek B., Hrstka R., Murray E., Gray T., and Hupp T. (2013) Emerging roles for the pro-oncogenic anterior gradient-2 in cancer development. Oncogene 32, 2499–2509 22945652 10.1038/onc.2012.346

[51] Baldauf H. M., Pan X., Erikson E., Schmidt S., Daddacha W., Burggraf M., Schenkova K., Ambiel I., Wabnitz G., Gramberg T., Panitz S., Flory E., Landau N. R., Sertel S., Rutsch F, Lasitschka F., Kim B., König R., Fackler O. T, and Keppler O. T. (2012) SAMHD1 restricts HIV-1 infection in resting CD4(+) T cells. Nat. Med. 18, 1682–1687 22972397 10.1038/nm.2964 PMC3828732

[52] Scherle P., Behrens T., and Staudt L. M. (1993) Ly-GDI, a GDP-dissociation inhibitor of the RhoA GTP-binding protein, is expressed preferentially in lymphocytes. Proc. Natl. Acad. Sci. U S A 90, 7568–7572 8356058 10.1073/pnas.90.16.7568 PMC47183

[53] Moissoglu K., McRoberts K. S., Meier J. A., Theodorescu D., and Schwartz M. A. (2009) Rho,GDP dissociation inhibitor 2 suppresses metastasis via unconventional regulation of RhoGTPases. Cancer Res. 69, 2838–2844 19276387 10.1158/0008-5472.CAN-08-1397 PMC2701105

[54] Cho H. J., Baek K. E., Park S. M., Kim I. K., Choi Y. L., Cho H. J., Nam I. K., Hwang E. M., Park J. Y., Han J. Y., Kang S. S., Kim D. C., Lee W. S., Lee M. N., Oh G. T., Kim J. W., Lee C. W., and Yoo J. (2009) RhoGDI2 expression is associated with tumor growth and malignant progression of gastric cancer. Clin. Cancer Res. 15, 2612–2619 19351766 10.1158/1078-0432.CCR-08-2192

[55] Went P. T., Lugli A., Meier S., Bundi M., Mirlacher M., Sauter G., and Dirnhofer S. (2004) Frequent EpCam protein expression in human carcinomas. Hum. Pathol. 35, 122–128 14745734 10.1016/j.humpath.2003.08.026

[56] Kimura H., Kato H., Faried A., Sohda M., Nakajima M., Fukai Y., Miyazaki T., Masuda N., Fukuchi M., and Kuwano H. (2007) Prognostic significance of EpCAM expression in human esophageal cancer. Int. J. Oncol. 30, 171–179 17143526

[57] Maetzel D., Denzel S., Mack B., Canis M., Went P., Benk M., Kieu C., Papior P., Baeuerle P.A., Munz M., and Gires O. (2009) Nuclear signalling by tumour-associated antigen EpCAM. Nat. Cell Biol. 11, 162–171 19136966 10.1038/ncb1824

[58] Rao C. G., Chianese D., Doyle G. V., Miller M. C., Russell T., Sanders R. A. Jr, and Terstappen L. W. (2005) Expression of epithelial cell adhesion molecule in carcinoma cells present in blood and primary and metastatic tumors. Int. J. Oncol. 27, 49–57 15942643

[59] Thorek D. L., Ulmert D., Diop N. F., Lupu M. E., Doran M. G., Huang R., Abou D. S., Larson S. M., and Grimm J. (2014) Non-invasive mapping of deep-tissue lymph nodes in live animals using a multimodal PET/MRI nanoparticle. Nat. Commun. 5, 3097 24445347 10.1038/ncomms4097 PMC4080716

[60] Driemel C., Kremling H., Schumacher S., Will D., Wolters J., Lindenlauf N., Mack B., Baldus S. A., Hoya V., Pietsch J. M., Panagiotidou P., Raba K., Vay C., Vallböhmer D., Harréus U., Knoefel W. T., Stoecklein N. H., and Gires O. (2014) Context-dependent adaption of EpCAM expression in early systemic esophageal cancer. Oncogene 33, 4904–4915 24141784 10.1038/onc.2013.441

[61] Zhang B., Wang J., Wang X., Zhu J., Liu. Q., Shi Z., Chambers M. C., Zimmerman L. J., Shaddox K. F., Kim S., Davies S. R., Wang S., Wang P., Kinsinger C. R., Rivers R. C., Rodriguez H., Townsend R. R., Ellis M. J., Carr S. A., Tabb D. L., Coffey R. J., Slebos R. J., Liebler D. C., and NCI CPTAC (2014) Proteogenomic characterization of human colon and rectal cancer. Nature 513, 382–387 25043054 10.1038/nature13438 PMC4249766

[62] Blakeley P., Overton I. M., and Hubbard S. J. (2012) Addressing statistical biases in nucleotide-derived protein databases for proteogenomic search strategies. J. Proteome Res. 11, 5221–5234 23025403 10.1021/pr300411q PMC3703792

[63] Seimetz D., Lindhofer H., and Bokemeyer C. (2010) Development and approval of the trifunctional antibody catumaxomab (anti-EpCAM x anti-CD3) as a targeted cancer immunotherapy. Cancer Treat Rev. 36, 458–467 20347527 10.1016/j.ctrv.2010.03.001

[64] Sakamoto J., Oriuchi N., Mochiki E., Asao T., Scott A. M., Hoffman E. W., Jungbluth A. A., Matsui T., Lee F. T., Papenfuss A., Kuwano H., Takahashi T., Endo K., and Old L. J. (2006) A phase I radioimmunolocalization trial of humanized monoclonal antibody huA33 in patients with gastric carcinoma. Cancer Sci. 97, 1248–1254 17034367 10.1111/j.1349-7006.2006.00324.x PMC11158047

[65] Chong G., Lee F. T., Hopkins W., Tebbutt N., Cebon J. S., Mountain A. J., Chappell B., Papenfuss A., Schleyer P., U P, Murphy R., Wirth V., Smyth F. E., Potasz N., Poon A., Davis I. D., Saunder T., O'keefe G. J., Burgess A. W., Hoffman E. W., Old L. J., and Scott A. M. (2005) Phase I trial of 131I-huA33 in patients with advanced colorectal carcinoma. Clin. Cancer Res. 11, 4818–4826 16000579 10.1158/1078-0432.CCR-04-2330

[66] Quoix E., Lena H., Losonczy G., Forget F., Chouaid C., Papai Z., Gervais R., Ottensmeier C., Szczesna A., Kazarnowicz A., Beck J. T., Westeel V., Felip E., Debieuvre D., Madroszyk A., Adam J., Lacoste G., Tavernaro A., Bastien B., Halluard C., Palanché T., and Limacher J. M. (2016) TG4010 immunotherapy and first-line chemotherapy for advanced non-small-cell lung cancer (TIME): results from the phase 2b part of a randomised, double-blind, placebo-controlled, phase 2b/3 trial. Lancet Oncol. 17, 212–223. 26727163 10.1016/S1470-2045(15)00483-0

[67] Vansteenkiste J., Zielinski M., Linder A., Dahabreh J., Gonzalez E. E., Malinowski W., Lopez-Brea M., Vanakesa T., Jassem J., Kalofonos H., Perdeus J., Bonnet R., Basko J., Janilionis R., Passlick B., Treasure T., Gillet M., Lehmann F. F., and Brichard V. G. (2013) Adjuvant MAGE-A3 Immunotherapy in Resected Non-Small-Cell Lung Cancer: Phase II Randomized Study Results. J. Clin. Oncol. 31, 2396–2403 23715567 10.1200/JCO.2012.43.7103

[68] Yoo C., Zhao J., Pal M., Hersberger K., Huber C. G., Simeone D. M., Beer D. G., and Lubman D. M. (2006) Automated integration of monolith-based protein separation with on-plate digestion for mass spectrometric analysis of esophageal adenocarcinoma human epithelial samples. Electrophoresis 27, 3643–3651 16927349 10.1002/elps.200600117

[69] Langer R., Ott K., Specht K., Becker K., Lordick F., Burian M., Herrmann K., Schrattenholz A., Cahill M. A., Schwaiger M., Hofler H., and Wester H. J. (2008) Protein expression profiling in esophageal adenocarcinoma patients indicates association of heat-shock protein 27 expression and chemotherapy response. Clin. Cancer Res. 14, 8279–8287 19088045 10.1158/1078-0432.CCR-08-0679

[70] Quaas A., Baha A.S., von Loga K., Seddiqi A.S., Singer J.M., Omidi M., Kraus O., Kwiatkowski M., Trusch M., Minner S., Burandt E., Stahl P., Wilczak W., Wurlitzer M., Simon R., Sauter G., Marx A., and Schlüter H. (2013) MALDI imaging on large-scale tissue microarrays identifies molecular features associated with tumour phenotype in oesophageal cancer. Histopathology 63, 455–462 23855813 10.1111/his.12193

[71] Aichler M., Elsner M., Ludyga N., Feuchtinger A., Zangen V., Maier S.K., Balluff B., Schöne C., Hierber L., Braselmann H., Meding S., Rauser S., Zischka H., Aubele M., Schmitt M., Feith M., Hauck S. M., Ueffing M., Langer R., Kuster B., Zitzelsberger H., Höfler H., and Walch A. K. (2013) Clinical response to chemotherapy in oesophageal adenocarcinoma patients is linked to defects in mitochondria. J. Pathol. 230, 410–419 23592244 10.1002/path.4199

[72] Elsner M., Rauser S., Maier S., Schöne C., Balluff B., Meding S., Jung G., Nipp M., Sarioglu H., Maccarrone G., Aichler M., Feuchtinger A., Langer R., Jütting U., Feith M., Küster B., Ueffing M., Zitzelsberger H., Höfler H., and Walch A. (2012) MALDI imaging mass spectrometry reveals COX7A2, TAGLN2 and S100-A10 as novel prognostic markers in Barrett's adenocarcinoma. J. Proteomics 75, 4693–4704 22365974 10.1016/j.jprot.2012.02.012

[73] Streitz J. M., Madden M. T., Marimanikkuppam S. S., Krick T. P., Salo W. L., and Aufderheide A. C. (2005) Analysis of protein expression patterns in Barrett's esophagus using MALDI mass spectrometry, in search of malignancy biomarkers. Dis. Esophagus 18, 170–176 16045579 10.1111/j.1442-2050.2005.00488.x

