# Supplementary material for: Quantitative Shotgun Proteomics Unveils Candidate Novel Esophageal Adenocarcinoma (EAC)-specific Proteins
Source: Mol Cell Proteomics. 2017 Jun;16(6):1138–50. doi: 10.1074/mcp.M116.065078 (PMC5461543; doi:10.1074/mcp.M116.065078)
Supplement: Supplemental Data [file supp_16_6_1138__index.html]

Quantitative Shotgun Proteomics Unveils Candidate Novel Esophageal Adenocarcinoma (EAC)-specific Proteins — Identifying Esophageal Adenocarcinoma-specific Proteins — Supplemental Data 

# Quantitative Shotgun Proteomics Unveils Candidate Novel Esophageal Adenocarcinoma (EAC)-specific Proteins

## Supplemental Data

- All\_quantitation (.xlsx, 1.2 MB) - Protein identifications and relative quantitation.
- Supplemental Data (.pdf, 1.4 MB)
