# Supplementary material for: Quantitative Shotgun Proteomics Unveils Candidate Novel Esophageal Adenocarcinoma (EAC)-specific Proteins
Source: Mol Cell Proteomics. 2017 Jun;16(6):1138–50. doi: 10.1074/mcp.M116.065078 (PMC5461543; doi:10.1074/mcp.M116.065078)
Supplement: Supplemental Data [file 10.1074_M116.065078_mcp.M116.065078-2.pdf]

SUPPLEMENT

SUPPLEMENTARY METHODS

The weight of a protein expression value (w) for one experiment was defined as;

$$w = \frac{1}{\sigma^2}$$

Equation 1 Weighting used for the contribution of each experiment to the mean. σ^2 = the variance in peptide ratios.

The weighted mean was then calculated as;

$$\overline{X} = \frac{\sum_{i=1}^{k} w_i x_i}{\sum_{i=1}^{k} w_i}$$

Equation 2 Calculation of the Weighted Mean. K=total number of experiments the protein was quantified in. X_i = the expression in experiment i. W_i = the weight for experiment i (see Equation 1).

The variance of the weighted mean was estimated as;

$$\sigma^2 = \frac{1}{\sum_{i=1}^{k} w_i}$$

Equation 3 Variance of the Weighted Mean

Calculation of Welch's t statistic

The Welch's t statistic was calculated for each protein for the difference between the mean TvE or TvG ratio and pooled mean TvT and EvE or TvT and GvG ratios.

$$t = \frac{\overline{X_1} - \overline{X_2}}{\sqrt{\frac{\sigma_1^2}{n_1} + \frac{\sigma_2^2}{n_2}}}$$

Equation 4 Welch's t statistic. X_1 = mean TvE or TvG ratio, X_2= mean TvT and EvE or TvT and GvG ratios, or for the pilot experiment the technical replicate ratio. σ^2 = variance, n=number of peptides contributing to the mean ratio. Derived from (1.

The degrees of freedom were calculated for each protein using Equation 5 and t tables were then used to calculate corresponding p-values on a per protein basis.

$$\nu = \frac{\left(\frac{\sigma_1^2}{n_1} + \frac{\sigma_2^2}{n_2}\right)^2}{\frac{\sigma^4}{n_1^2(n_1 - 1)} + \frac{\sigma^4}{n_2^2(n_2 - 1)}}$$

Equation 5. Calculation of the degrees of freedom for Welch's t test (from (2)).

SUPPLEMENTARY TABLES

Table S1. Clinical characteristics of cohort used for the EAC tissue microarray.

		Mean	Number of Patients	Percentage (%)
Age (years)		63.9		
Gender	Female		18	15.7%
	Male		97	84.3%
Histology	ACC		115	100.0%
Surgery	Yes		115	100.0%
Neoadjuvant Chemotherapy	No		86	74.8%
	Yes		29	25.2%
Operation Type	ETG		1	0.9%
	Transhiatal		8	7.0%
	Ivor-Lewis		81	70.4%
	LTA		25	21.7%
Maximum tumour diameter (mm)		45.3		
Differentiation	Well		2	1.7%
	Moderate		47	40.9%
	Poor		66	57.4%
Resection Status	R0		46	40.0%
	R1		69	60.0%
T Stage (TNM 7)	T1b		8	7.0%
	T2		12	10.4%
	T3		90	78.3%
	T4a		5	4.3%
Nodal Stage (TNM 7)	N0		29	25.2%
	N1		23	20.0%
	N2		29	25.2%
	N3		34	29.6%
Follow-up	Alive		9	7.8%
	Dead		106	92.2%

ETG – Extended total gastrectomy, LTA – Left thoracoabdominal gastrectomy, ACC - Adenocarcinoma

Table S2. Optimised IHC staining conditions for validation candidates

Antibody, source, reference	Antigen retrieval (citric acid buffer)	Blocking conditions	Primary dilution of manufacturer stock
AGR2, Sigma, HPA007912	20 mins WB	H_2O_2 10 mins then SFPB 20 mins	1/1000 in diluent
SAMHD1, Abcam, ab119751	5 mins PC	H_2O_2 10 mins then SFPB 20 mins	1/600 in diluent
HSPA5, Abcam ab21685	20 mins WB	H_2O_2 10 mins then SFPB 20 mins	1/200 in diluent
ARHGDIB, Abcam ab88317	20 mins WB	H_2O_2 10 mins then SFPB 20 mins	1/600 in diluent
EpCAM, Abcam, ab187372	20mins WB	H_2O_2 10 mins	1/500 in diluent
HSPB1, Abcam ab114067	No AR	H_2O_2 10 mins then SFPB 20 mins	1/1000 in diluent
TGM3, Sigma, HPA004728	5 mins PC	H_2O_2 10 mins then SFPB 20 mins	1/300 in diluent
Citric Acid buffer – (1.8 mM Citric Acid, 8.2 mM Sodium Citrate in dH_20, pH 6.0)<br>WB – Water Bath (98ºC), PC – Pressure Cooker, AR – Antigen Retrieval, H_2O_2 - 3% (v/v) Hydrogen Peroxide, SFPB – Serum Free Protein Block (DAKO, Carpinteria, CA)			

Table S3. Relative expression and significance levels for verification candidates.

Gene Name	Entrez GeneID	Median Normalised Log2 TvE	FDR-corrected P (TvE)	Median-normalised Log2 TvG	FDR-corrected P (TvG)	Peptides quantified	Number of Replicates Observed In
ARHGDIB	397	**0.283**	2.09E-62	**0.782**	3.58E-91	52	8
SAMHD1	25939	**0.157**	1.52E-78	**0.409**	2.20E-143	130	14
AGR2	10551	**0.526**	4.75E-104	**-0.459**	0	778	14
HSPA5	3309	**0.284**	0	**-0.403**	0	1992	12
EPCAM	4072	**2.964**	2.03E-14	**3.646**	8.27E-26	10	4
TGM3	7053	**-1.374**	0	**0.246**	6.85E-44	642	14
HSPB1	3315	**-1.028**	0	**0.611**	0	1050	13

SUPPLEMENTARY REFERENCES

1. Welch, B. (1947) The generalization of "Student's" problem when several different population variances are involved. *Biometrika.* **34**, 28-35.
2. Satterthwaite, FE. (1946) An approximate distribution of estimates of variance components. *Biometrics Bulletin.* **2**, 110-114

SUPPLEMENTARY FIGURE LEGENDS

Supplementary Figure 1. *The distribution of median-normalised, log_2 protein expression ratios. (A) Histograms. Note differing x-axis (log) scales. (B) Boxplots of the same data. Boxplot whiskers represent 1.5 interquartile ranges above or below the 75th and 25th percentiles respectively. Unfilled circles represent outlier points.*

Supplementary Figure 2. *Validation of the sensitivity and specificity of an anti-ARHGDIB antibody. (A) Lysates from each of the denoted oesophageal cell lines, labelled with the corresponding cell type, were resolved by SDS-PAGE and western blots probed with antibodies to ARHGDIB (green) or β-Actin (red) before secondary incubation with a fluorophore-conjugated antibody and scanning using a fluorescent imaging system. ESCC – Esophageal Squamous Cell Carcinoma. (B) To confirm antibody specificity, scrambled siRNA, siRNA to ARHGDIB or vector were transfected into OE33 cells and a western blot performed using cell lysates, 72 hours after transfection.*

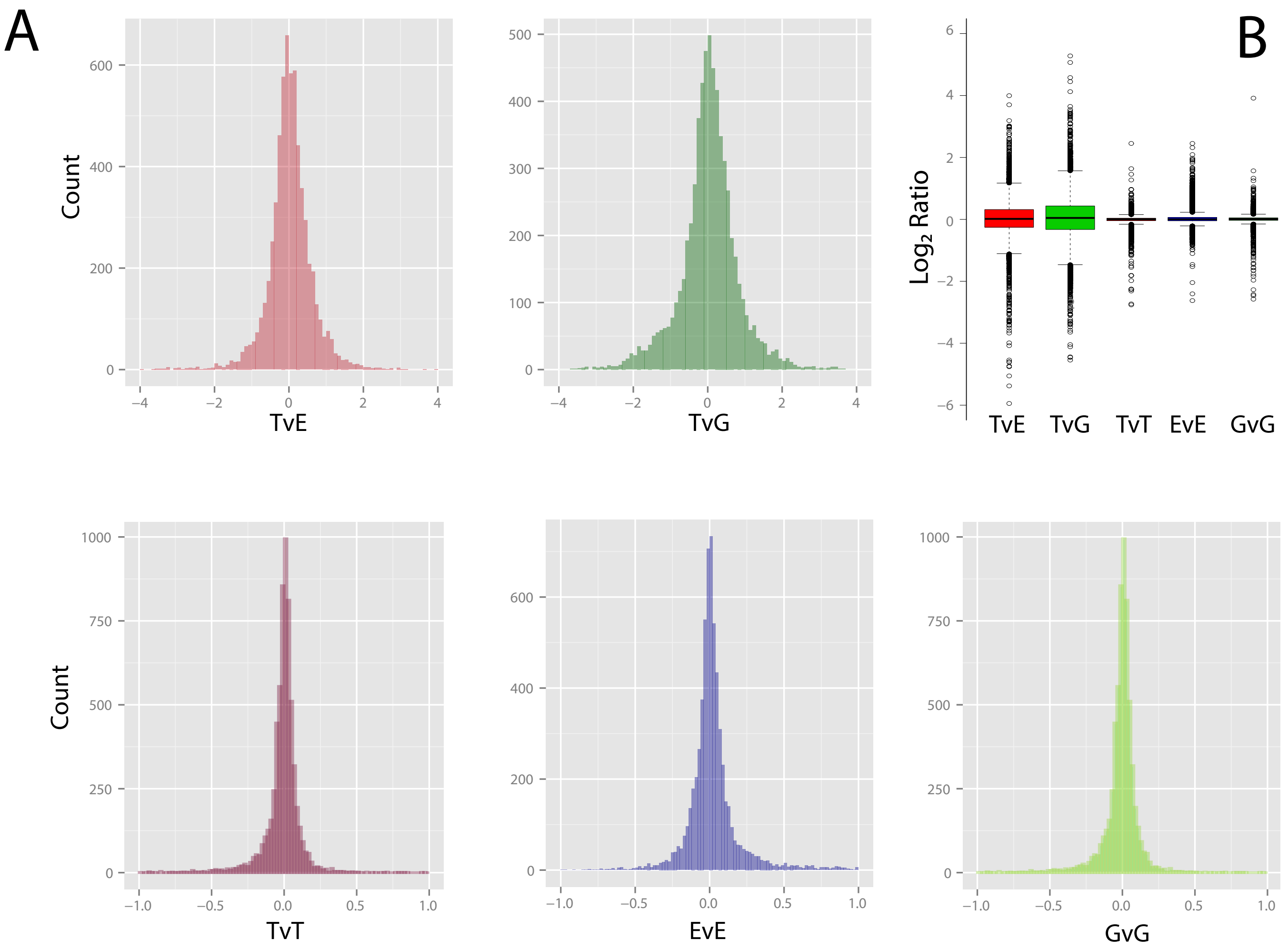
A
B
Count
TvE
TvG
Log2 Ratio
TvE
TvG
TvT
EvE
GvG
Count
TvT
EvE
GvG

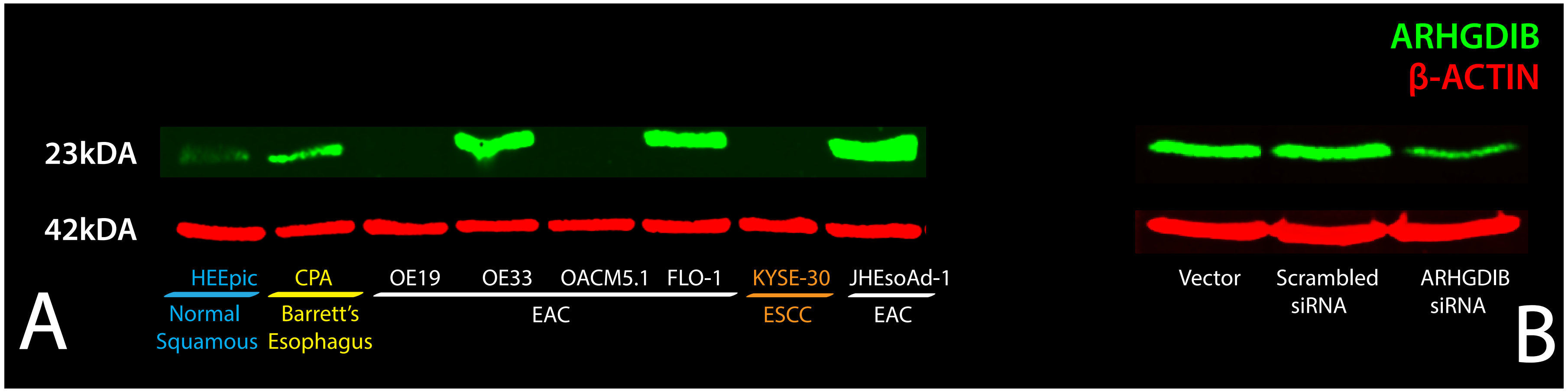
ARHGDIB
β-ACTIN
23kDA
42kDA
HEEpic
CPA
OE19
OE33
OACM5.1
FLO-1
KYSE-30
JHEsoAd-1
Normal Squamous
Barrett's Esophagus
EAC
ESCC
EAC
A
Vector
Scrambled siRNA
ARHGDIB siRNA
B
